# A Novel Robust Smart Energy Management and Demand Reduction for Smart Homes Based on Internet of Energy

**DOI:** 10.3390/s21144756

**Published:** 2021-07-12

**Authors:** Bilal Naji Alhasnawi, Basil H. Jasim, Zain-Aldeen S. A. Rahman, Pierluigi Siano

**Affiliations:** 1 Electrical Engineering Department, Basrah University, Basrah 61001, Iraq; bilalnaji11@yahoo.com (B.N.A.); hanbas632@gmail.com (B.H.J.); as.zain9391@stu.edu.iq (Z.-A.S.A.R.); 2 Department of Electrical Techniques, Qurna Technique Institute, Southern Technical University, Basra 61016, Iraq; 3 Management and Innovation Systems Department, Salerno University, 84084 Fisciano, SA, Italy; 4 Department of Electrical and Electronic Engineering Science, University of Johannesburg, Johannesburg 2024, South Africa

**Keywords:** dynamic electricity price, optimization algorithms, home devices, Internet of things, MQTT protocol

## Abstract

In residential energy management (REM), Time of Use (ToU) of devices scheduling based on user-defined preferences is an essential task performed by the home energy management controller. This paper devised a robust REM technique capable of monitoring and controlling residential loads within a smart home. In this paper, a new distributed multi-agent framework based on the cloud layer computing architecture is developed for real-time microgrid economic dispatch and monitoring. In this paper the grey wolf optimizer (GWO), artificial bee colony (ABC) optimization algorithm-based Time of Use (ToU) pricing model is proposed to define the rates for shoulder-peak and on-peak hours. The results illustrate the effectiveness of the proposed the grey wolf optimizer (GWO), artificial bee colony (ABC) optimization algorithm based ToU pricing scheme. A Raspberry Pi3 based model of a well-known test grid topology is modified to support real-time communication with open-source IoE platform Node-Red used for cloud computing. Two levels communication system connects microgrid system, implemented in Raspberry Pi3, to cloud server. The local communication level utilizes IP/TCP and MQTT is used as a protocol for global communication level. The results demonstrate and validate the effectiveness of the proposed technique, as well as the capability to track the changes of load with the interactions in real-time and the fast convergence rate.

## 1. Introduction

Internet of Energy (IoE) plays a significant role in today’s world through promoting social and economic development. A Wireless Sensor Network (WSN) is considered the key technology in IoE architecture, which plays a significant role in promoting IoE. The IoE is now extensively used in various fields such as smart cities, healthcare, smart power grids, etc. [1,2].

The development of a loE of systems assists Home Energy Management System (HEMS) to monitor different home devices based on the collected information of the devices with different wireless technologies. The Wi-Fi platform for HEMS is included in this paper. Wi-Fi is a wireless network with standard number IEEE 802.15.4 from the Electrical and Electronics Engineers Institute. The main purpose of Wi-Fi is to design digital low-power signal systems with a low bandwidth (PAN) network [3]. Wi-Fi technology can assist HEMS in real-time collecting the energy information to help the users to provide smart and efficient management on the smart home devices. According to the aforementioned cases, home devices interact together through a wireless network for optimal and efficient monitoring by the aim of HEMS.

In order to ensure an intelligent grid and to enhance the IoE integration, continuous communication is necessary. In terms of output, market, distribution, transmission and customer the intelligent grid is more effective than a conventional grid [4]. The conventional grids have limited power generation, and a large number of small power producers are in the intelligent grid. A conventional grid transmission includes large power lines and pipelines with a smart grid that offers small transmission and compensation for local supply, thereby making the smart grid significantly more efficient than the conventional grid. Consumers of a smart grid are very active and participate in a system in the form of priorities and demands set. The national and centralized market for a conventional grid is fragmented and the borders are ignored. A smart grid consists of billions of intelligent devices, sensors, intelligent meters and many other communication networks, be they private or public [5,6].

Demand response (DR) strategies provide economic, effective and secure solutions in relation to energy management. Energy management system (EMS) on the DR decentralized residential or housing microgrid offers the most cost-effective demand profile, so that load control will help the house owners have less discomfort [7]. However, installing a collocated roof generator and the variability of home appliances in the operation of the storage system can make it difficult to manage the energy of a building process. This is more demanding as it is quite difficult to estimate renewable real-time generation and also since the energy price in the day ahead is different from the real-time energy price. Stochastic optimization may address these uncertainties, but it also requires appropriate random parameter probabilistic estimates. For domestic energy management, the real-time optimization portfolio is therefore required, which can provide an optimal solution in the abrupt change in energy generation and the price of market energy. To fulfil the abovementioned requirement, this paper elucidates a real-time optimization system for synergetic source load storage dispatch in an intelligent home or an intelligent residential microgrid.

Conventionally, control has been implemented as a single centralized controller, in which all the energy nodes are connected to one another and to the central control unit through bidirectional communication links to gather system-wide information. These communication links increase the cost and decrease the reliability of the MG. Centralized control ensures low-voltage performance through capability, accurate power sharing, etc., but is prone to a single point of failure; that is, if the central controller or one of the communication links fails, it causes the entire MG to collapse. Furthermore, this strategy places a large computational burden on the central controller, thus making its design complex and costly.

The decentralized control method comprises several individual controllers that need only local measurements, but it does not require a high-bandwidth communication infrastructure (except for synchronization purpose). Thus, it is proven to be more reliable than the centralized strategy because of its limited communication infrastructure. However, due to the lack of system-wide information, all the available energy sources in an MG cannot be efficiently harmonized in an optimum way [8].

To counteract the stated limitations of the two control strategies, the distributed control method has been proven to be very reliable; it is influenced by the idea of a multi-agent system (MAS). In this method, the energy nodes are considered to be agents that can exchange information with their neighboring nodes through a sparse communication infrastructure. Consequently, the cost of the communication infrastructure decreases and the system reliability increases [9]. Table 1 shows the main aspects of the control techniques.

Therefore, this paper, a distributed method is proposed based on a Multi-agent System (MAS) algorithm.

The rest of this study is organized as follows: Section 2 provides Literature review of Theoretical background, Section 3 provides proposed system description, Section 4 presents experimental results validation, Section 5 presents results discussion, Section 6 presents the conclusions of the paper.

## 2. Literature Review of Theoretical Background

This section describes some recent literature pertinent to energy management in microgrid. Table 2 involves the contributions and shortcomings of the most recent research applied energy management system in the.

### 2.1. Research Gaps

From the literature, many essential research gaps have been identified.

1. In many systems such as [10,15,16,17,18] The energy management for a multi-agent system governed microgrid in Energy Internet not investigated.
2. In many systems such as [30,31,32,33] a cloud-based platform for energy management system in microgrid not investigated.
3. In many systems such as [22,24,25], the authors did not use a meta-heuristic technique (the grey wolf optimizer, artificial bee colony optimization algorithm, etc.) to minimize the cost.
4. In some of the above studies such as [26,28] the focus of the authors is on approach to solve energy management problem, However, the transfer of a massive amount of data on the existing communication infrastructure is challenging.
5. In many papers, the user comfort and PAR are ignored, which are directly linked with the total electricity bill.
6. In some of the above studies such as [11,12,13] the authors reduced peaks in demand while user comfort is minimized.

### 2.2. Paper Contribution

In this paper, a novel real-time electricity scheduling for smart demand side management system using the IoT is proposed that employs: scalability, adaptability, Interoperability and connectivity between appliances over cloud platforms. The key contributions of this paper are summed up here:

- Firstly, the researchers investigated the MAS-controlled MGs in the Energy Internet, which has not been reported in the past.
- Proposed an advanced demand management scheme based on the grey wolf optimizer (GWO), artificial bee colony (ABC) optimization algorithm to minimize power mismatching, energy bill and load energy waste.
- Thirdly, they implemented a framework for the proposed control technique using MAS and cloud servers.
- Furthermore, we proposed an IoT-based communication protocol, which included specifications such as MQTT. This improves system flexibility. The proposed system offered analytics and business intelligence (BI), which allowed the researchers to gain insights on the data collected by visualizing dashboards and reports. Additionally, the use of big data-based data storage technologies enabled the system’s scalability at the national level. This provided energy-efficiency strategies for the household owners and the utility companies.
- We implemented a hierarchical two-layered communication architecture based on the MQTT protocol and using the cloud-based server called Node-Red. This helped customers realize the global and local communications necessary for the neighborhood appliance controllers.

## 3. Methodology

### 3.1. Proposed System Description

**Figure 1:**
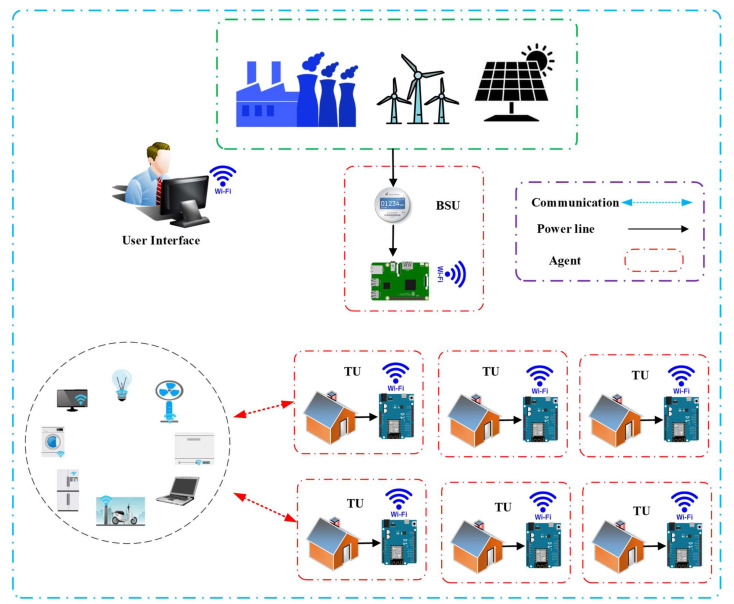
Proposed system.

Here, the researchers considered that the DGs consisted of the communication and control agents in the Internet of Energy realm, as described in Figure 1. It was noted that the DGs in microgrid were controlled by a framework, wherein every DG was managed by one MAS (Multi agent System). The MAS agents communicate by Local Area Network (LAN) and can access the internet for remotely controlling the micro grid via the cloud servers. In the Energy Internet, every distributed generator/microgrid was managed by various stakeholders, and their controllers on the MAS/agents differed from MG components. Figure 1 presents the proposed system.

Smart grid would need an effective measuring and communication system in order to continuously track the power and cost profile and regularly quantify power losses. There are several stages of data processing.

This work contains measurement units (MU) for every Distribution Network Bus. MU is MATLAB modeling. Power and cost information is sent to the control center regularly at fixed time. The control center is designed as a virtual data management and analysis platform. One approach to communication relating to the device topology proposed is considered. The case takes a Cloud approach, which sends its measured data directly to the Cloud by any MU connected to corresponding feeder bus as illustrate in Figure 1.

Real-time data transfer among the Raspberry Pi3 package and the open source IoT framework Node-Red are used to model proposed communication architectures. Node-Red was chosen for the simulation of real time cloud communication Due to its following benefits [34]:

1. Node-Red Cloud IoT platform data aggregation, tracking and analysis. In the smart grid model, power profile is monitored on multiple Node-Red channels in real time and depicted graphically.
2. Security: The Username and password allow user authentication while each channel is equipped with its own ID and can be accessible (see by other users). There are two keys in each channel for the application programming interface. A randomly generated read key and write key of the API. These keys can save or retrieve information from stuff from each channel over the Internet or LAN.
3. It facilitates the double-way flow of data between user and virtual device and allows data and remote control to be exchanged in real time. The MATLAB Desktop Real-time Toolbox offers a communication between the simulated feeding model and the Node-Red IoT platform.
4. Communication network enabling for real-time data transmission over the Internet between Raspberry Pi3 and Node-Red.
5. Allows importing, exporting, analyzing and viewing data on multiple platforms and their fields simultaneously.

### 3.2. Problem Formulation

We consider a home energy management system that equips a household with a single power management system and various types of appliances to reduce its energy consumption. A two-way communication network that enables price exchange and information about the energy consumption in accordance with applied conditions is the energy management system connected to the supplier. The energy management system receives the information on the hourly price of the service provider and manages the energy conversion in response to the price levels of each device.

Electric household appliances are generally divided into three main types according to their features and imports, containing non-shiftable, shiftable and controllable loads. The following paragraphs detail the mathematical wording of the home energy management system counting the numerous operating constraints for all appliance groups and the objective function.

To minimize the objective function of cost of devices in individual and community consumer:

(1) $$minP_{n}=\underset{n\in N}{\sum }\underset{t\in T}{\sum }\underset{q\in Q}{\sum }\underset{d\in D}{\sum }(\alpha_{qd}^{n,t}\times L_{qd}^{n,t}\times P_{E}^{n,t}-\beta_{r}^{n,t}\times S_{r}^{n,t}\times P_{r}^{n,t}+\gamma_{P}^{n,t}\times L_{L}^{n,t}\times P_{P}^{n,t})$$

where, $\gamma_{P}^{n,t}$ is the decision flexible, N is the total amount of users, T is the time, Q is the type of load, D is the total number of devices, $\alpha_{qd}^{n,t}$ is the decision variable for the appliances, $P_{E}^{n,t}\;$is the electricity, $S_{r}^{n,t}$ is the decision variable for energy, $L_{L}^{n,t}$ is the electricity storage at time t, $L_{qd}^{n,t}\;$is the power profile of the house devices [35].

#### 3.2.1. Non-Shiftable Appliances

Non-shifting appliances require critical demands that have to be fulfilled during the energy distribution process, such as certain security alarm framework. The non-shiftable load starts to work, it must work constantly and cannot be programmed. The energy use of these appliances always meets energy demand [36].

(2) $$E_{n,h}^{\mathrm{non}\;}=e_{n,h}^{\mathrm{non}\;}$$

where $n\in \left\{1,\;2,\;3,\ldots ,N\right\}$ indicate a device n, N is total number of devices, $h\in \left\{1,\;2,\;3,\ldots ,\;H\right\}\;$represents an hour and H is a final day hour. $E_{n,h}$ and $e_{n,h}$ represents the actual energy consumption and electricity demand of devices n at hour h, respectively. The costs of such devices are only the energy consumption bill for electricity. So, a non-shiftable utility function device n is:

(3) $$U_{n,h}^{\mathrm{non}}=P_{h}\cdot E_{n,h}^{\mathrm{non}}$$

where $P_{h}$ represent price at h.

#### 3.2.2. Shiftable Appliance

Shifting loads can plan their demand for energy to off-peak hours if the price is low on the horizon, not only prevents the maximum energy consumption but also reduce the energy bill. Shift able appliances have two operating points available, ‘off’ and ‘on’.

(4) $$E_{n,h}^{\mathrm{shift}}=I_{n,h}\cdot e_{n,h}^{\mathrm{shift}\;}$$

where $I_{n,h}$ is a binary variable for device n, i.e., $I_{n,h}=1$ if device works at h; otherwise $I_{n,h}=0$.

There are two types of costs for this type of device: the electricity bill for energy consumption and the lack of satisfaction with waiting times to start and then conclude the operation of the device. For instance, during the work period, the washing equipment (WM) usually operates (i.e., 18–23 pm), but time can be changed from high price electricity to low price periods, if the WM starts to work at $T_{n,w}$, in this case, the waiting time would be $T_{n,w}-T_{n,\mathrm{ini}}$ (i.e., 3 h).

Shiftable utility function [36]:

(5) $$U_{n,h}^{\mathrm{shift}\;}=P_{h}\cdot E_{n,h}^{\mathrm{shift}\;}+k_{n}\cdot \left(T_{n,w}-T_{n,\mathrm{ini}}\right)$$

(6) $$T_{n,\;\mathrm{ini}\;}\leq T_{n,w}\leq \left[T_{n,\;\mathrm{end}\;}-T_{n,\mathrm{ne}}\right]$$

(7) $$T_{n,\mathrm{ne}}\leq T_{n,\;\mathrm{end}\;}-T_{n,\;\mathrm{ini}\;}$$

where electricity costs represent the first term and the second term refers to waiting time costs in Equation (5). $K_{n}$ is a system-dependent coefficient, $T_{n,\;\mathrm{ini}\;}$ is initial time and $T_{n,\;\mathrm{end}\;}$ is end time, $T_{n,w}$ represent the operation starting time and $T_{n,\mathrm{ne}}$ indicates the time required for the shiftable devices.

#### 3.2.3. Modeling of Energy Storage

In order to achieve green energy goals and ensure system reliability, the energy storage system plays a vital role. Therefore, an energy storage system is used for storing excess available energy in our considered home energy management system. $E_{b}\left(t\right)$ indicates the energy stored in the battery at any time, t and given in Equation (8). $E_{b}\left(t\right)$. has a positive loading value, while the discharge is negative. η^c^ and $\eta^{d}$ denote to charging and discharging efficiencies of the battery. The constraints given in Equations (9) and (10) are considered for limiting the maximum charging and discharging states of energy storage system. $\delta_{b}\left(t\right)$ is a binary variable at time t [37].

(8) $$E_{b}\left(t\right)\&=\frac{E_{b}^{c}\left(t\right)}{\eta^{c}}\delta_{b}\left(t\right)-E_{b}^{d}\left(t\right)\eta^{d}\left(1-\delta_{b}\left(t\right)\right)$$

(9) $$0\&\leq \frac{E_{b}^{c}\left(t\right)}{\eta^{c}}\leq E_{b,\max}^{c}\left(t\right)\delta_{b}\left(t\right)$$

(10) $$0\&\leq E_{b}^{d}\left(t\right)\eta^{d}\leq E_{b,\max}^{d}\left(t\right)\left(1-\delta_{b}\left(t\right)\right)$$

(11) $$\mathrm{SOC}\left(t\right)=\mathrm{SOC}\left(t-1\right)+\frac{E_{b}\left(t\right)}{C_{b}}$$

(12) $${\mathrm{SOC}}_{\max}\leq \mathrm{SOC}\left(t\right)\leq {\mathrm{SOC}}_{\min}$$

The state of charge (SoC) of battery is modeled in Equations (11) and (12) models the minimum and maximum SoC limits of battery at time t. The battery rated capacity is denoted by $C_{b}$.

#### 3.2.4. Modeling of Photovoltaic

This study model uses the probabilistic models of beta distribution-based solar irradiance and photovoltaic generation variability. The probabilistic model of solar irradiance random variable G is obtained by describing it in probability density function (pdf) expression as,

(13) $$f\left(G\right)=\frac{\Gamma \left(a+b\right)}{\Gamma \left(a\right)\Gamma \left(b\right)}\Gamma^{a-1}{\left(1-\Gamma \right)}^{b-1},0<G<\infty$$

where $f\;\left(G\right)$ is the probabilistic beta distribution function of solar irradiance random variable G. a and b are the parameters, which are calculated using mean G and standard deviation $\sigma_{G}^{2}$ of random variable G.

(14) $$b=\left(1-\mu_{G}\right)\times \left(\frac{\left(\mu_{G}\left(1+\mu_{G}\right)\right)}{\sigma_{G}^{2}}-1\right)$$

(15) $$a=\frac{{b\mu }_{G}}{1-\mu_{G}}$$

The power generated by the photovoltaic system after inverter, $P_{\mathrm{pv}}\;$at time t in the suggested home energy management system is determined by Equations (16) and (17):

(16) $$P_{\mathrm{pv}}\left(t\right)\&=\eta_{\mathrm{pv}}\eta_{\mathrm{inv}\;}P_{\mathrm{pv},n}\frac{G\left(t\right)}{G_{\mathrm{stc}}}\left[1-\lambda \left(T_{c}\left(t\right)-T_{\mathrm{stc}}\right)\right]$$

(17) $$T_{c}\left(t\right)\&=T_{a}+\frac{G\left(t\right)}{G_{\mathrm{NOCT}}}\left(\mathrm{NOCT}-20\right)$$

where $P_{\mathrm{pv},n}$, $\eta_{\mathrm{pv}}$ and $\eta_{\mathrm{inv}\;}$ are rated power of photovoltaic panel, photovoltaic panel efficiency and inverter efficiency, respectively. The values of $\eta_{\mathrm{pv}}$ and $\eta_{\mathrm{inv}}$ are 92% and 95%, respectively. λ is temperature-dependent power degradation coefficient. $T_{c}\left(t\right)$ is photovoltaic cell temperature at time t. $G_{stc}$ and $T_{stc}$ are solar irradiance and temperature at standard temperature condition (stc). NOCT is nominal operating cell temperature [37].

#### 3.2.5. Preference of Operation Period

The binary matrix is utilized for a ready-to-use factor. This requires the ready-to-use slot $w_{qd}^{n,t}$ to run the devices over time. Home users tend to operate a computer more often throughout the day, and then substitute it with more devices.

(18) $$P_{1}:\alpha_{qd}^{n,t}=\alpha_{qd}^{n,t}\times w_{qd}^{n,t}$$

#### 3.2.6. Variable Decision

Constraint $P_{2}$ is the decision variable of the device ON/OFF. Constraints $P_{3}$ is decision variable of user for self generation power. If $\beta_{r}^{n,t}=1$, user is a prosumer and $\beta_{r}^{n,t}=0\;$for user is a consumer. Consumers purchase electricity from the neighborhood microgrid or power grid.

(19) $$P_{2}:\alpha_{qd}^{n,t}\in \left\{0,1\right\}\;\;\;\;\;\;\;\;\;\;\;\forall q,\;t\in T$$

(20) $$P_{3}:\beta_{r}^{n,t}\in \left\{0,1\right\}\;\;\;\;\;\;\;\;\;\;\;\forall q,\;t\in T$$

(21) $$P_{4}:\gamma_{P}^{n,t}\in \left\{0,1\right\}\;\;\;\;\;\;\;\;\;\;\;\forall q,\;t\in T$$

#### 3.2.7. Devices Task

For the measurement of energy profiles, it is mandatory to know the working life of intelligent devices.$\;t_{qd}\;$is the operation time of $d_{th}$ devices in the T slot time in $P_{5}$. $\alpha_{qd}^{n,t}$ is the decision variable to turn ON/OFF the device. The constraints $P_{5}$ and $P_{6}$ are continuous times to accomplish a task and it has to remain ON at time T, until it has finished a task. For instance, once a washing machine begins to work, it runs continuously until the final time limit is set, $P_{6}\;$is formulated. ts is the devices starting time [35].

(22) $$P_{5}:\overset{T}{\underset{t=1}{\sum }}\alpha_{qd}^{n,t}=t_{qd}\;\;\;\;\;\;\;\;\;\;\;\;\;\;\;\;\;\;\;\;\;\;\;\;\;\;\;\;\;\;\;\;\;\;\;\;\;\;\;\forall q,\;t\in T$$

(23) $$P_{6}:\overset{ts+tqd-1}{\underset{t=ts}{\sum }}\alpha_{qd}^{n,t}=t_{qd}\;\;\;\;\;\;\;\;\;\;\;\;\;\;\;\;\;\;\;\;\;\;\;\;\;\;\;\;\;\;\;\forall q,\;t\in T$$

#### 3.2.8. Devices Priority

When another system completes the service cycle, the appliance will start running. A dryer will not operate until the laundry has completed its operating cycle. $s_{i}$ is the group of these kind of loads. The decision variable selects the devices of each group for each time span.

(24) $$P_{7}:\underset{d\in s_{i}}{\sum }\alpha_{qd}^{n,t}=1\;\;\;\;\;\;\;\;\;\;\;\;\;\;\;\;\;\;\;\forall q,\;t\in T$$

#### 3.2.9. Price

The price signal is received from the group micro grid. A utility grid is optional for our study, the energy volume for export and import from the community. The dynamic-pricing system is used for electronic transactions from the grid. The prices are believed to be accepted and cannot be updated after publication. The pricing system can be freely chosen by the customers. The costs of the same load will differ at different times in one day. The electricity is consistently low and expensive to obtain from the grid at night and vice-versa. The energy price depends on the energy used and the time per day the energy is used.

(25) $$P_{E}^{n,t}=\left\{\begin{array}{cc} P_{r}=0.3 & if\;\;r_{sa}=1\\ P_{b}=0.7 & if\;E_{ba}=1\\ P_{g}>P_{b}>P_{r} & O.W \end{array}\right\}$$

where $P_{E}^{n,t}$ is the electricity tariff, $P_{r}$ and $P_{b}$ are electricity prices from the community micro grid and $P_{g}$ is a utility grid purchase.

#### 3.2.10. Energy Transaction with Grid

The home management system imports energy from the main grid in case of local energy depletion and exports energy to the main grid in case of local excess energy availability. The total energy transaction is calculated using the main grid Equation (26)

(26) $$E_{tr}\left(t\right)=E_{ha}\left(t\right)-E_{pv}\left(t\right)+E_{b}\left(t\right)$$

where $E_{tr}\left(t\right)$, $E_{pv}\left(t\right)$ and $E_{b}\left(t\right)$ are the total energy transacted with main grid, photovoltaic energy generation and the batteries charge (discharge) energy at time *t*, respectively. A positive of $E_{b}\left(t\right)$ represent the charging of the batteries and negative value of $E_{b}\left(t\right)$ represent the discharging state of the batteries [37].

#### 3.2.11. Multi Agent System (MASs) Communication

**Figure 2:**
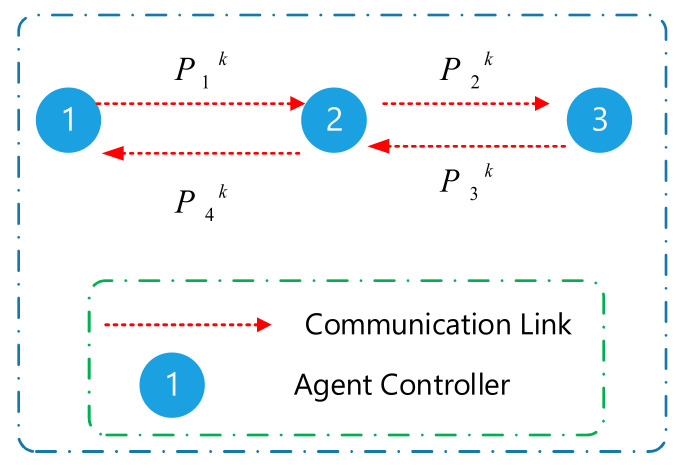
Exchange information among agents.

The communication networks of microgrid having N agents were represented using a graph: $G=\left(P_{G},E_{G}\right)$ having a defined set of nodes $P_{G}=\left\{p_{1},p_{2},\;\ldots ,\;p_{N}\right\}$ and edges $E_{G}\subseteq P_{G}\times P_{G}$. All nodes presented in the graph G(agents) showed a one-to-one correspondence to the nodes in the graph T(DGs). Furthermore, the edges in G, which represented the communication links for the data exchange, differed from the electrical connection seen in T. In addition, the set of neighbors described in the *i^th^* node of G was represented by $N_{i}=\left\{p_{j}\in P_{G}:\left(p_{i},p_{j}\right)\in E_{G}\right\}$. The researchers represented the adjacency matrix as $\left[a_{ij}\right]\subseteq R^{n\times n}.$ Here, the term $a_{ij}$ represented the information that was exchanged between the agents i and j, wherein $a_{ij}=1$ when agents i and j were connected with the edge ($p_{i},p_{j})\in E_{G}$, else $a_{ij}=0$. The researchers represented the Laplacian matrix as $L=\left[l_{ij}\right]\subseteq R^{n\times n}$ where each element $l_{ij}=\sum_{i=1}^{n}a_{ij}-a_{ji}$. They described the pinning matrix as $G=diag\left[g_{i}\right]\subseteq R^{n\times n}$ and $g_{i}=1$ when the agent could access the references $P^{ref}$ else $g_{i}=0.$ Figure 2 presents an example of the data exchange between the controllers.

### 3.3. Proposed Communication Platform of Energy Internet

**Figure 3:**
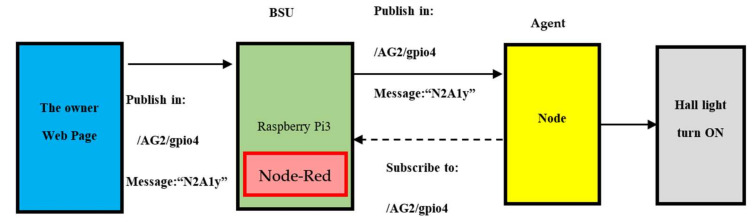
Proposed simulation Internet of Energy platform architecture.

The decentralised controller of a smart MG helps in managing the system operating conditions if there is some disturbance. Furthermore, the IoT technology can be used for communicating between the appliances present in smart homes, central controller or power management centres. The researchers proposed the IoT platform for collecting the data, monitoring, managing and controlling microgrid. All appliances and energy resources were integrated and connected in this platform. Proposed internet of energy communication platform presented in Figure 3.

It is a demanding job to develop an energy management distributed Energy Internet (IoE) base. The role of the platform is to (1) incorporate the micro-grid tools into the communications system and (2) link to the IoE cloud in order to track and manage the devices. The IoE communications network proposed is composed of 4 different layers, as defined in Figure 2.

#### 3.3.1. The MQTT Knowledge

The MQTT is a lightweight protocol. The MQTT is operational in the TCP and ensures that all messages are forwarded from agent to server.

**Figure 4:**
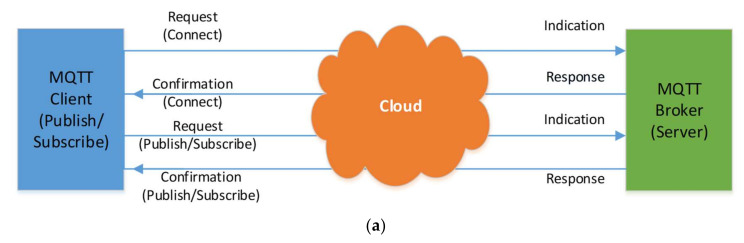

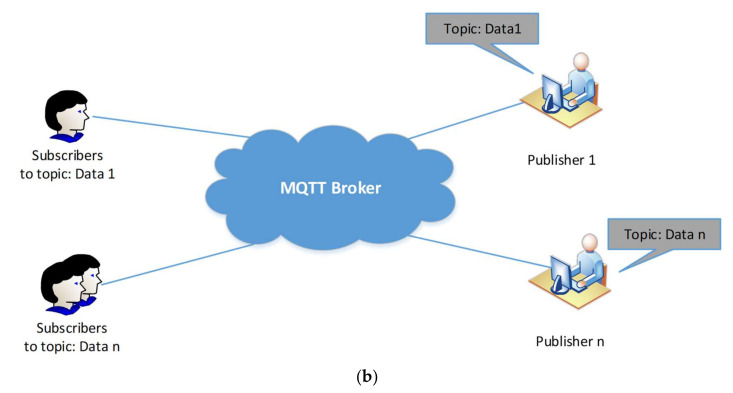
(**a**) MQTT Procedure, (**b**) MQTT Topic and Component.

Three major players, i.e., A MQTT protocol includes MQTT Publisher, MQTT Broker and a MQTT subscriber. MQTT’s subscribing and publishing companies have indirect connections and no IP address at the same time. An MQTT broker takes care of the customer authorization and initialization process necessary for communication. To publish the information, the MQTT publishers utilize custom themes for catering to their clients. The MQTT protocol did not use a Metadata marking. Thereafter, the MQTT topic management presents the metadata for a message load. MQTT is known as a string with the hierarchical structure of multi-attribute and multi levels. Every stage can be separated from the forward slash in a theme tree [38]. For routing data derivation, all subjects can be modified. Following the exchange of control packets among clients and brokers, Figure 4a presents the link initialization. Check packets for CONNAC, Connect, PUBACK, PUBLISH, SUBSCRIBE, SUBACK, etc., were shown to include specific instructions concerning the subject, transmission and payload service quality (QoS). Figure 4b presents all components of the MQTT contact.

#### 3.3.2. Proposed Architecture

**Figure 5:**
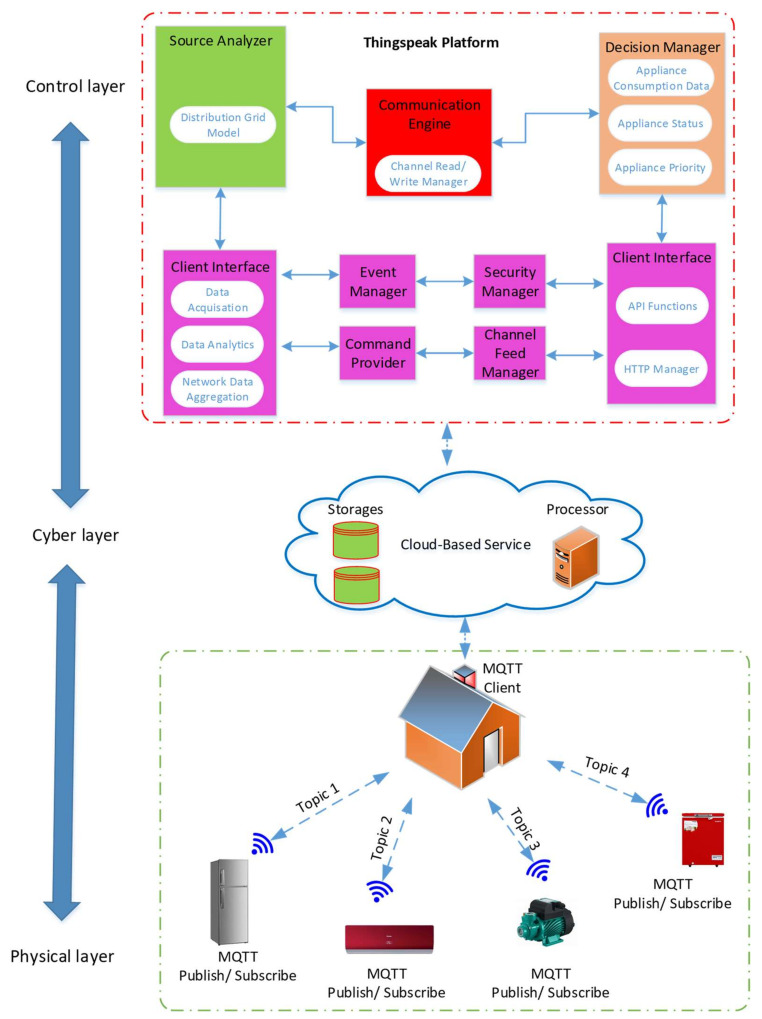
Smart home proposed architecture of communication.3.4. Proposed Optimization Method.

The hierarchical system provided for intelligent homes with a control-layer and cyber-layer is presented in Figure 5. 2 communication layers were included in the hybrid platform. It was seen that in the Layer 1 (local layer), the appliances in the smart building transmitted the MQTT messages to a Building MQTT Client (BMC) and reported the measurement and subscribed to the MQTT message that were published via MQTT Client for protection/control purpose. A connection between the Cloud and BMC using HTTP POST/GET requests was seen in Layer 2 (who is the global layer). Any appliance in this architecture had Wi Fi unit connected to the local gate way. Thus, values of a committed and pre-defined subject could be published regularly [39,40]. The BMC then subscribes to the various topics and transmits the values obtained to the cloud channel. A MATLAB cloud interface, which implements the built algorithm for allocating appliances, is available to access all cloud data. The algorithm results are then transferred with BMC from a cloud to intelligent devices that control them. The researchers found that when communication in any layer fails, the suggested architecture is robust (either local or global). Hence, the BMC was so designed that during any communication link failure or high latency noted in the network, it could operate as a local controller for all appliances in building. This function of BMC was highlighted in the Results section.

#### 3.3.3. Grey Wolf Algorithm

The proposed energy management strategy consists of three phases: input, process and out. The demand-side management strategy home energy management systems handle the input variables to calculate the total satisfactory desired day satisfaction values and provide the grey wolf satisfaction algorithm with all the input parameters calculated to identify the optimum scheduling pattern for the devices that provide the highest level of satisfaction, i.e., the output. The process of calculating the method designed is further elaborated.

##### The Objective Function

The objective function via grey wolf accretive satisfaction algorithm is the absolute satisfaction level, via generating a best scheduling pattern of house devices [41].

(27) $$\mathrm{Obj}\left(\mu_{s},C_{\beta }\right)=max\left(\mu_{s}\right)$$

That $C_{s\_\mathrm{index}\;}\left(\$\right)$ depends upon consumer satisfaction and total consumer expenses thus Grey Wolf accretive satisfaction algorithm cost function can also be described as

(28) $$\mathrm{Obj}\left(C_{s\_\mathrm{index}\;}\left(\$\right)\right)=min\left(C_{s_{-}\mathrm{index}\;}\left(\$\right)\right)$$

##### Constraints

Grey Wolf accretive satisfaction algorithm is subject to two constraints of energy consumption.

Budget constraint of Grey Wolf accretive satisfaction algorithm refer to as the total user electricity expenses $TU_{\exp}$ must be less than the already defined budget limit C of consumer which can be stated as:

(29) $$TU_{\exp}\leq C_{\beta }$$

(30) $$TU_{\exp}=TEC\times UT$$

(31) $$TEC=\overset{n=A}{\underset{Z}{\sum }}(TOT_{n}\times TPR_{n})$$

where $TOT_{n}$ is total operational time, $TPR_{n}$ represent total power rating.

**Figure 6:**
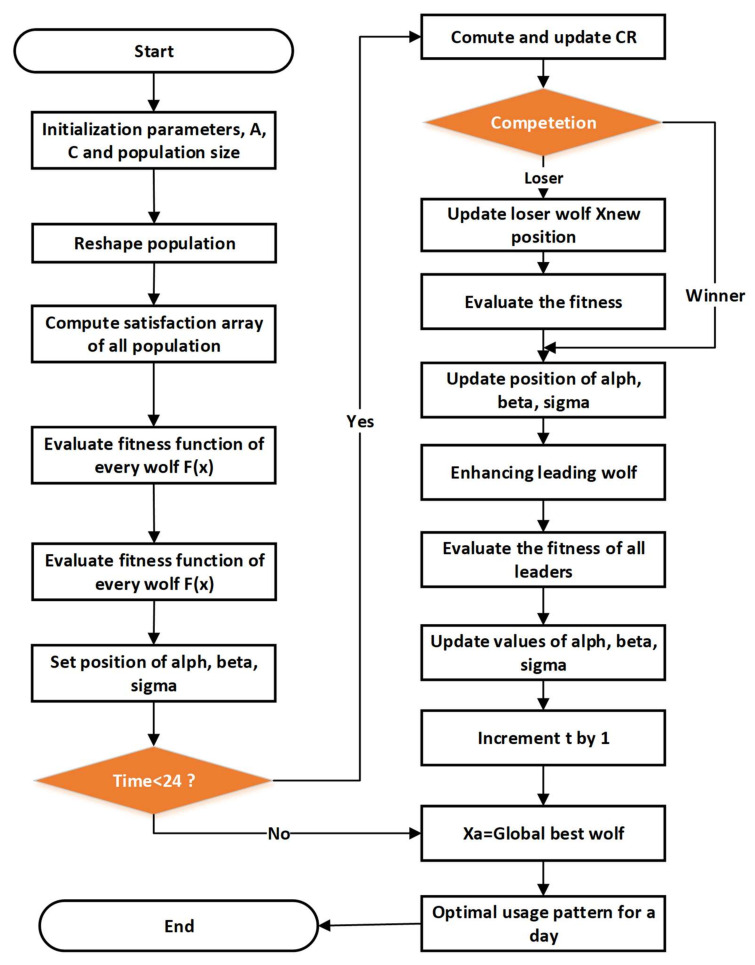
Flow diagram of grey wolf algorithm.

The maximum amount of energy available is the energy constraint that should not be infringed; the consumer can eat within one day. This is why energy can be restricted:

(32) TEC≤TAE

(33) $$TEA=\frac{C_{\beta }\left(\$\right)}{UT\left(\$/kWh\right)}$$

where total energy *TEA* is available to consumers as much as possible as their energy budget can be determined,

(34) $$X^{d}\left(t+1\right)=\left\{\begin{array}{cc} 1,\;\;\;\;\;\;\;\;\;\;\;\;\;\;\;S\left(X_{1}^{d}+X_{2}^{d}+X_{3}^{d}/3\right)⩾r_{8} & \\ 0, & \;\mathrm{otherwise}\; \end{array}\right.$$

(35) $$\begin{array}{c} X_{1}\;=\;\left|X_{\alpha }-2a\cdot r_{1}-a\cdot {\bar{D}}_{\alpha }\right|\\ X_{2}\;=\;\left|X_{\beta }-2a\cdot r_{1}-a\cdot {\bar{D}}_{\beta }\right|\\ X_{3}\;=\;\left|X_{\delta }-2a\cdot r_{1}-a\cdot {\bar{D}}_{\delta }\right| \end{array}$$

where $r_{1}$ belongs to a vector of [0, 1], $X_{1}^{d}$, $X_{2}^{d}$, $X_{3}^{d}$ are updated position at iteration t as described in Equation (35). A value of ${\bar{D}}_{\alpha }$, ${\bar{D}}_{\beta }$, ${\bar{D}}_{\delta }$ can be obtained from [41]. It is evident in the above equations that the loser learns to update their positions by the winners; therefore, the performance of the BGWO will increase. The following details are given as to the steps taken by GWASA to achieve an optimum scheduling pattern for device use. Figure 6 shows flowchart of grey wolf algorithm:

(36) $$\begin{array}{c} A=2a\times r_{1}-a\\ C=2r_{2}\\ a=2-2\left(t/T\right) \end{array}$$

(37) $$L^{a}=\left\{\begin{array}{cc} \mathrm{rand}\left(0,1\right) & \;\mathrm{if}\;\mathrm{CR}\;\leq r_{9}\\ X_{L}^{d}, & \;\mathrm{otherwise}\; \end{array}\right.$$

(38) $$\begin{array}{c} CR=0.9-0.9\left(t/T\right) \end{array}$$

#### 3.3.4. Artificial Bee Colony Algorithm

In 2005, Karaboga described the algorithm for bee swarms known by its name as Artificial Bee Colony (ABC). The chief idea is honey bee movements’ smart and behavior [42]. The best way of combating local extremes is to use a global algorithm such as ABC algorithm. The food in the colony is composed of three players: (i) sources of food; (ii) bees employed and (iii) unemployed bees divided to spectators and scout bees. The Employed bees look into a source of food and perform a negotiated dance to attract visitors to the food sources after returning to a colony. As the duration of dance is associated with the consistent supply of food, stronger suppliers (global Optima) are more likely to prefer onlooker bees. The used bee becomes a bee that looks unexpectedly for fresh food when the food supply is depleted.

The on-sight bee monitors and sends many employed bees to find the source of food. During each iteration, the scout bee offers the same solution. The viewing bee then checks on the best solution and saves it in memory for fitness (e.g., cost function). A viewer bee selects, after a certain number of iterations, the best optimal solution from multiple solutions. In phase two, an onlooker bee is directed to find the random source of food by an observer (i.e., random solutions for DG size). The scout bee is assigned the task for a global optimal solution in order to avoid trapping in local minima. to the random search.

The first factors are the amount of food dots (NFP), which equal the total number of bees, within the ABC algorithm. Random numbers are used to form the initial solution population, with the following random positions [42]:

(39) $$X_{ij}=X_{j,min}+\mathrm{rand}\times \left(X_{j,max}-X_{j,min}\right),\;i=1,2,\ldots NFP,j=1,2,\ldots ,J$$

where $X_{ij}\;$is the ith population and NFP is a set. $X_{j,min}\;$and $X_{j,max}\;$illustrate minimum and maximum boundaries of jth vector. Rand is at the same time a random number, distributed in a uniform way between 0 and 1. The following can show the fitness function:

(40) $${\mathrm{Fitness}}_{i}\;=\;\mathrm{Obj}(X_{ij})\;+\;\overset{M}{\underset{m=1}{\sum }}\;\lambda_{\mathrm{eq},m}{\left|h(X_{ij})\right|}^{2}\;+\;\overset{N}{\underset{n=1}{\sum }}\;\lambda_{\mathrm{ineq}\;,n}{\left|g(X_{ij})\;-\;g_{lim}\right|}^{2}$$

where Obj is the objective function, while equality and inequality constraints are represented by $h\left(X_{ij}\right)\;$ and $g\left(X_{ij}\right)$. The $\lambda_{eq,m}$ and $\lambda_{\mathrm{ineq}\;,n}$ can be adjusted in the optimisation process. glim can be represented as:

(41) $$g_{\lim}=\left\{\begin{array}{ll} X_{j}, & \;\mathrm{if}\;X_{j,min}\leq X_{j}\leq X_{j,max}\\ X_{j,min}, & \;\mathrm{if}\;X_{j}<X_{j,min}\\ X_{j,max}, & \;\mathrm{if}\;X_{j}>X_{j,max} \end{array}\right.$$

**Figure 7:**
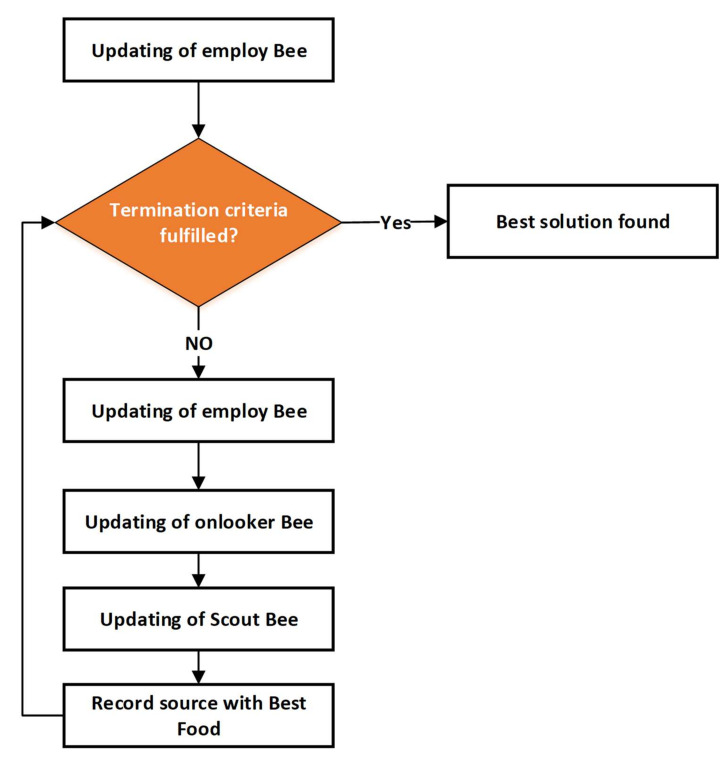
Flowchart of Artificial Bee Colony Algorithm.

When one or more variables violate the limits and person concerned is, therefore, discarded to skip the infeasible solution, value of penalty factor can be increased. Figure 7 illustrates the flowchart of the ABC algorithm.

## 4. Experimental Results Validation

To experiment and prove the benefits of proposed home energy management system over a cloud as a service (HEMaaS), many services have been evaluated and implemented over the platform.

**Figure 8:**
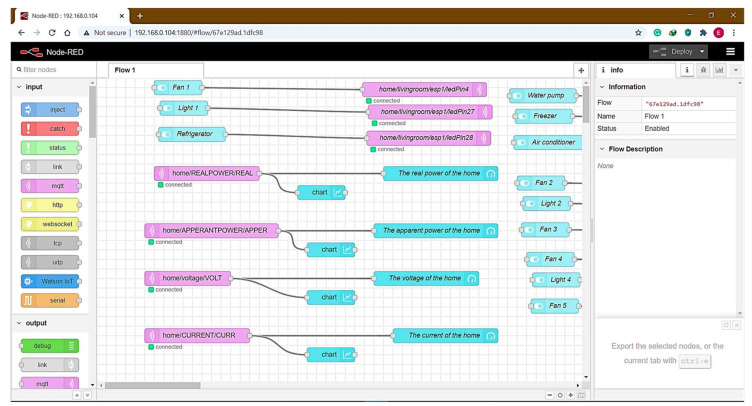
Node-Red platform.

In this section, HEMaaS results have been presented and discussed with the suggested algorithm through a platform cloud to regulate devices in the smart home. A Raspberry-Pi3 in the Main Command and Control Unit (MCCU) organizes node-red platforms, as developed in the Software Communication and Architecture Interface. As a broker between the home device subscription and the publisher of the MCCU Protocol. A custom python code using the suggested algorithm is used for regulating the house device operation via the MQTT Gateway. In this study, a user interface (UI) is designed with the Node-Red dashboard interface, allowing a customer to access and interact via a Cloud Service System with HEMaaS. The dashboard control design is illustrated in Figure 8.

### 4.1. The Base Station Unit

**Figure 9:**
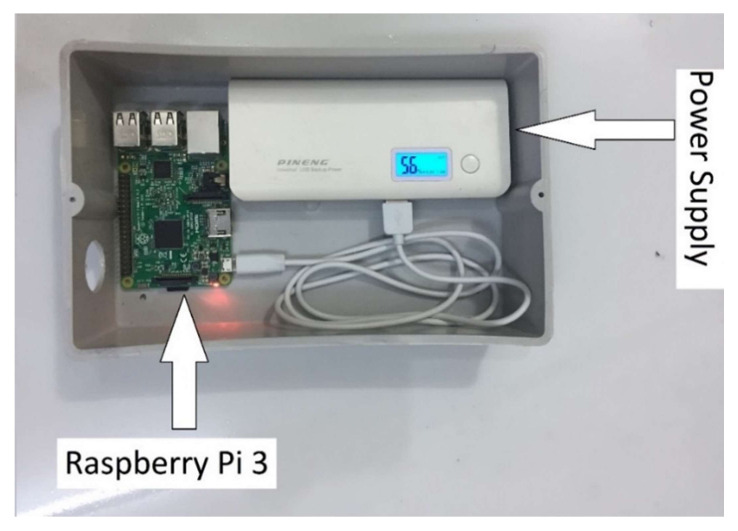
Base Station Unit structure.

In the proposed system the Base Station Unit (BSU) plays an important role. The BSU is the coordinator of the system. A Raspberry Pi3 board is a Base Station hardware unit. The Base Station Unit analyzes and transmits data to the mobile and Web page of the owner. To establish the Wi-Fi connection the terminal units can connect to this, the basic station unit should be set up in access point mode. Mosquito, an open-source message broker implementing the MQTT protocol, was installed in the Base Station Unit. MQTT offers a lightweight method to perform messaging using a publish/subscribe model, which consists of a 2-byte fixed-header method. Figure 9 shows the Base Station Unit.

### 4.2. Terminal Unit

The Terminal Units (TUs) are the sub-units of the Wireless Sensor Network (WSN) system. Each TU comprises the sensor, processor, wireless communication and power module.

**Figure 10:**
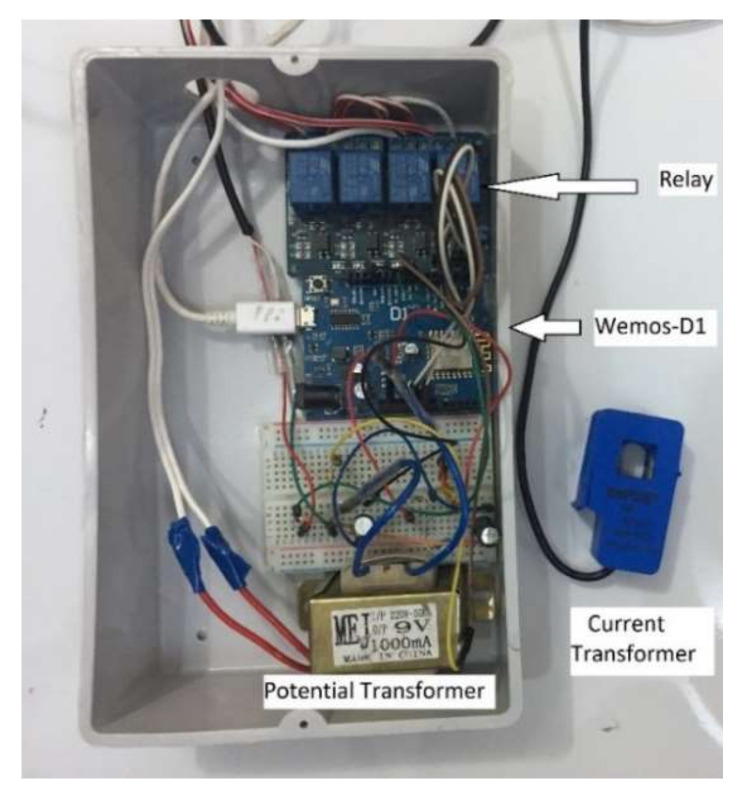
Terminal Unit.

**Figure 11:**
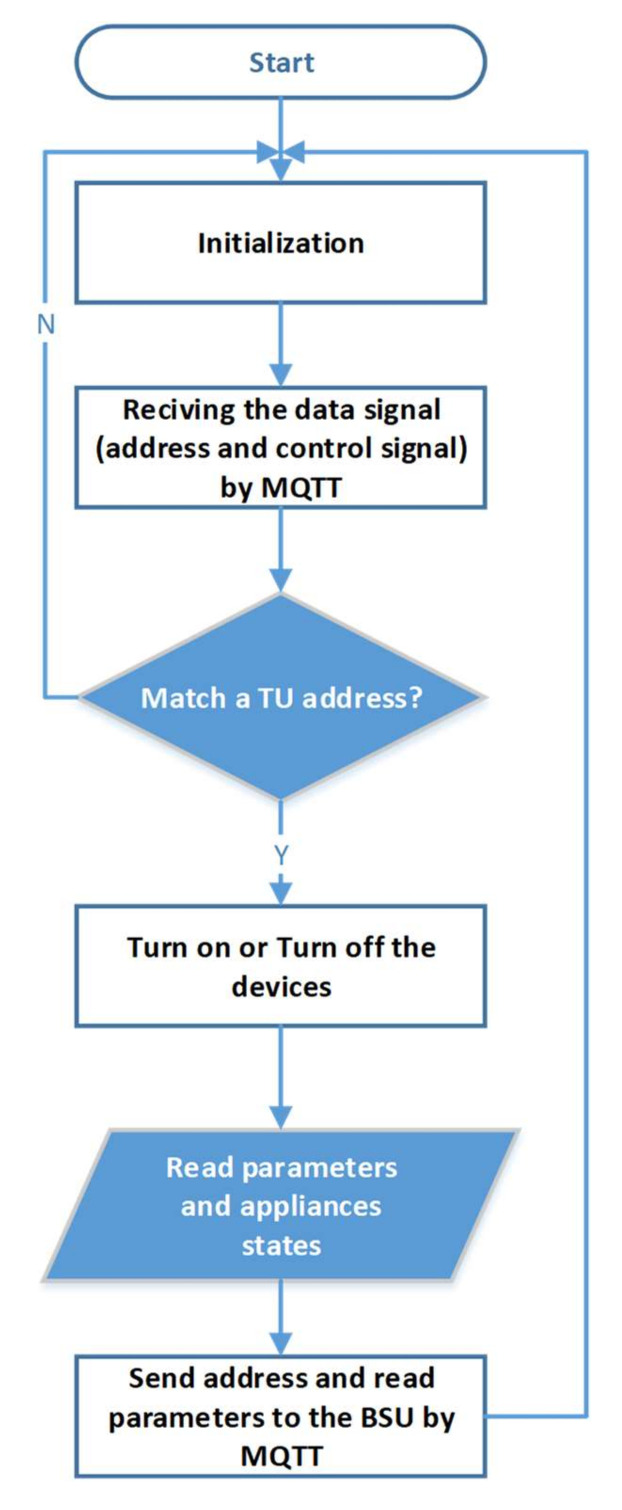
Terminal Unit flowchart.

The agent controller is a Wemos-D1 board that collects and processes sensor data and transmits the information obtained to the Base Station Unit. Figure 10 shows the internal structure of the prototype Terminal Unit used for implementing the system. Figure 11 illustrates a flowchart of the Terminal Unit.

### 4.3. Access of Internet Web Page

**Figure 12:**
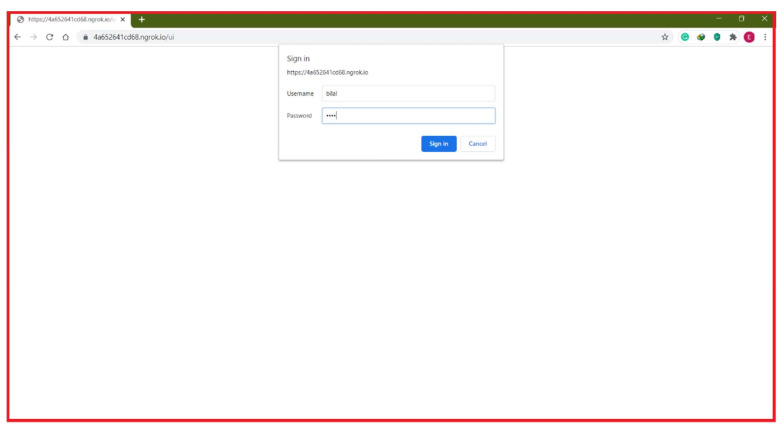
Web site to enter username and password in the browser.

To access the web page locally, Raspberry Pi3 IP local ports 1880 for the Node-Red site are used for internet protocol (IP). The local IP is http://192.168.0.104:1880/ui. The Ngrok server can convert the local IP address of Raspberry Pi3 from anywhere in the world to a global IP address. The web page http://4a652641cd68.ngrok.io, is accessible during Ngrok’s registration for the web page. Figure 12 shows the web page of a web browser after the username and password have been entered and provided in the URL.

For scheduling, where each house has several devices, three intelligent homes are envisaged. We considered the same energy demand for any consumer for a fair comparison of the electricity bill for three households. Table 3 includes a comprehensive overview of each apparatus. The appliances selected have three groups: no interruptible, interruptible, scheduled and non-schedulable.

**Figure 13:**
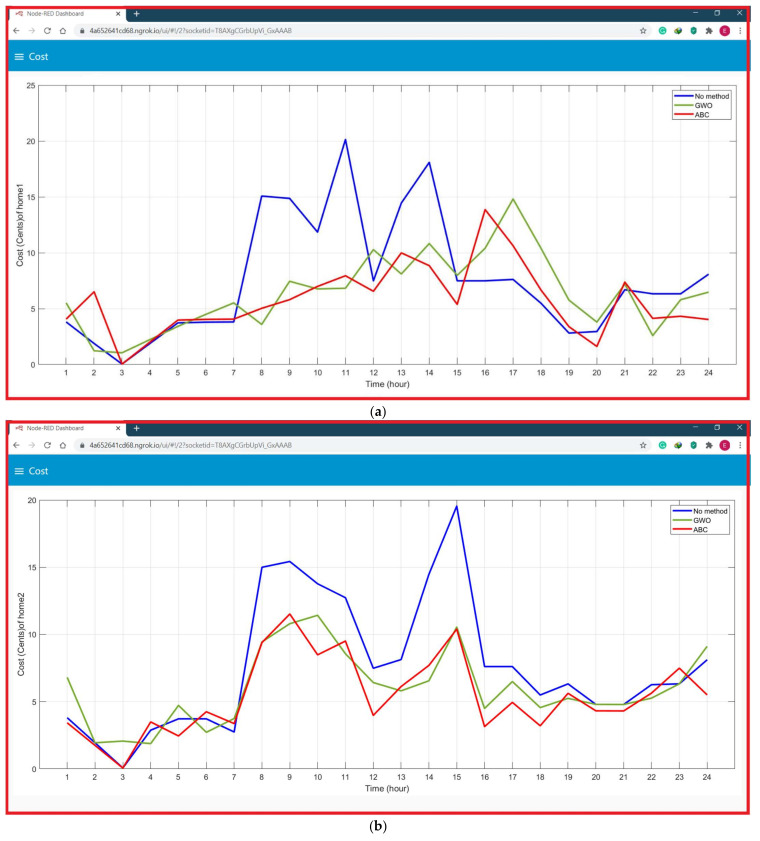

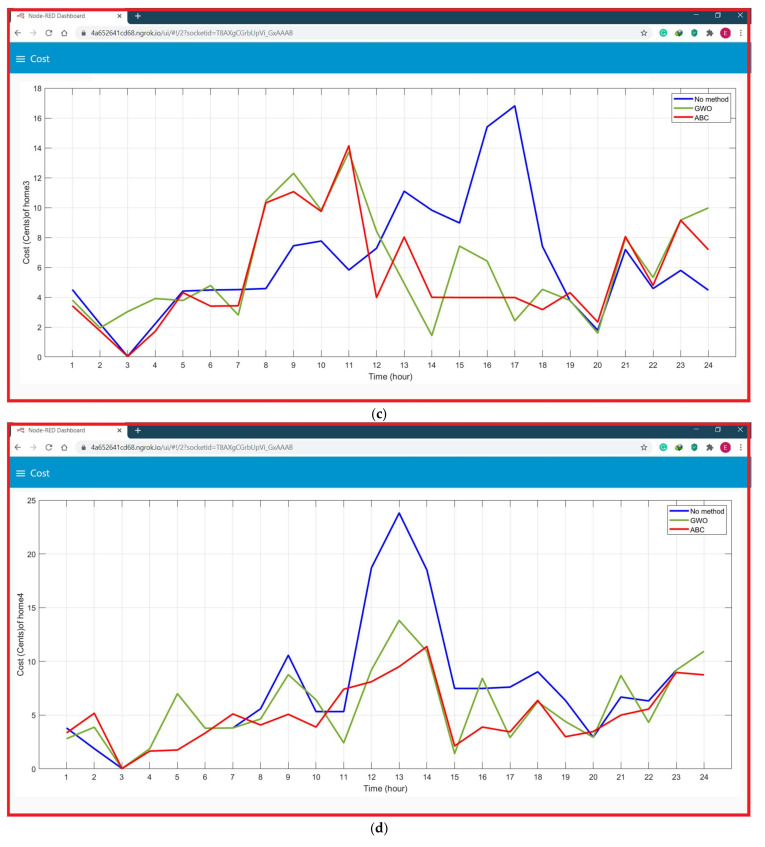

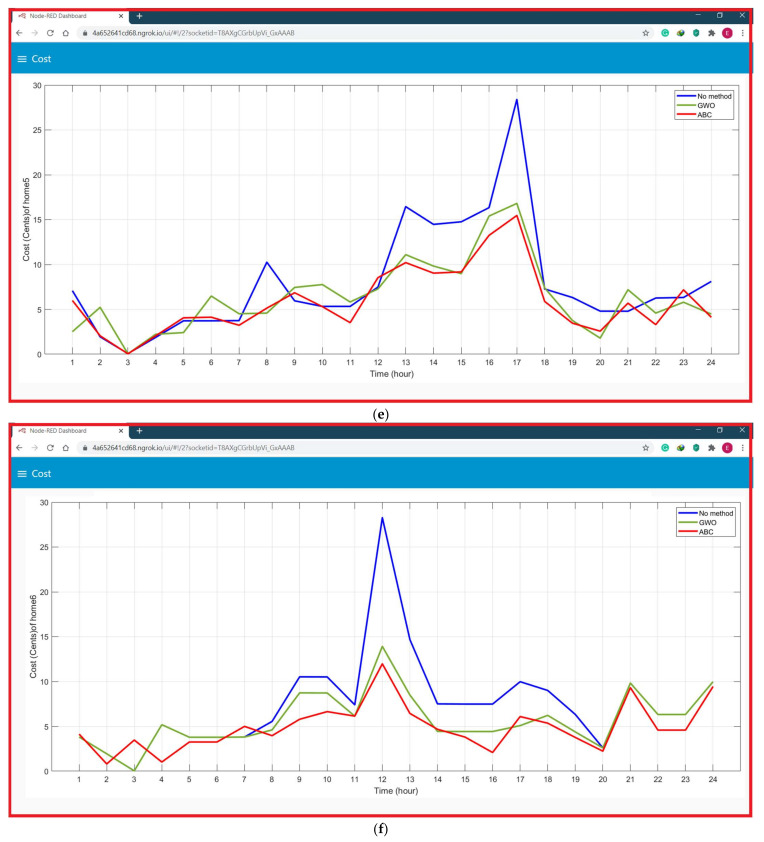
Node-RED dashboard GUI of proposed home EMS before and after implementing the GWO and ABC algorithms, (**a**) cost profiles of first house, (**b**) cost profiles of second house, (**c**) cost profiles of third house, (**d**) cost profiles of fourth house, (**e**) cost profiles of fifth house and (**f**) cost profiles of the sixth house.

The home energy management system comprises Graphical User Interface (GUI) and related software to facilitate users power consumption and total cost, of microgrid devices; their power consumption of all homes without corrective method is implemented as displayed in Figure 13 shows cost GUI of proposed home EMS before and after implementing the GWO and ABC algorithms, (a) cost profiles of first house, (b) cost profiles of second house, (c) cost profiles of third house, (d) cost profiles of fourth house, (e) cost profiles of fifth house and (f) cost profiles of the sixth house

## 5. Results Discussion

Electricity emission cost reduction and cost savings, as well as PAR, were examined in an efficiency analysis of the microgrid. The time slots required for non-shiftable and shiftable demand have been moved into the morning time following the application of the suggested energy management system, where the power prices of utilities are low, as shown in Figure 12. The load profile has therefore been corrected as shiftable customer devices that are not shiftable can operate at low prices in time slots. As a result, consumption energy costs have been reduced, emission costs decreased and PAR improved.

**Figure 14:**
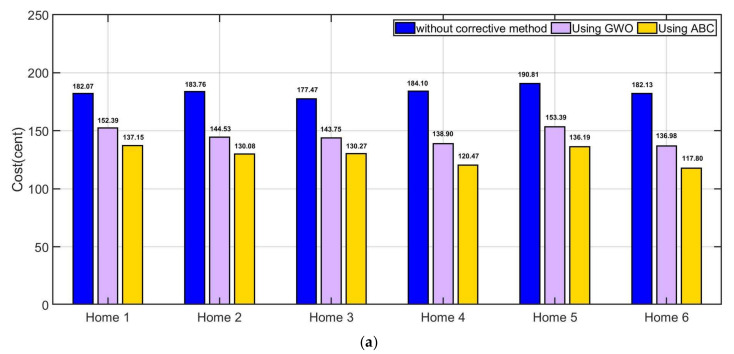

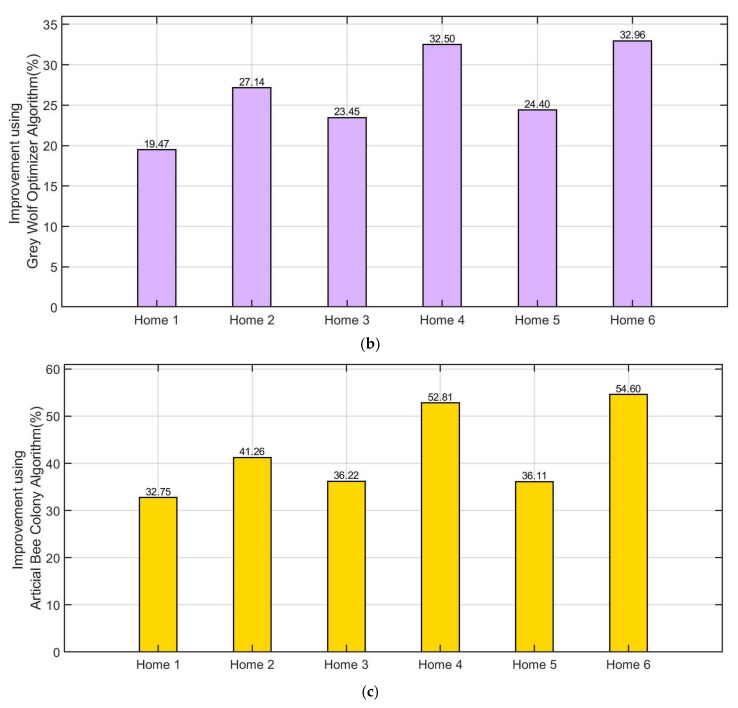
Comparison between without suggested EMS and with suggested EMS, (**a**) microgrid cost, (**b**) improvement (%) by using GWO and (**c**) improvement (%) by using ABC.

The price before applying the proposed algorithm is 182.07 for home 1, 183.76 for home 2 and 177.4667 for home 3, 184.0955 for home 4, 190.8146 for home 5 and 182.13 for home 6. Whereas after applying the proposed GWO, the cost of is found 152.386 for home 1, 144.5312 for home 2, 143.7466 for home 3, 138.9 for home 4, 153.3869 for home 5 and 136.98 for home 6 in case of weekday. Whereas after applying the proposed ABC, the cost of is found 137.1482 for home 1, 130.078 for home 2, 130.27 for home 3, 120.4749 for home 4, 136.19 for home 5 and 117.8039 for home 6 in case of weekday. By comparing proposed algorithms with traditional method, the suggested GWO method in our work saved 19.47% for home 1, 27.14% for home 2, 23.45% for home 3, 32.5% for home 4, 24.4% for home 5 and 32.96% for home 6 per-day. The suggested ABC method in our work saved 32.75% for home 1, 41.26% for home 2, 36.22% for home 3, 52.81% for home 4, 36.11% for home 5 and 54.6% for home 6 per-day. The comparison between with and without corrective method is illustrated in Table 4. Figure 14 shows the comparison between without suggested EMS and with suggested EMS.

## 6. Conclusions

In this study, a novel robust smart EMS and demand reduction for smart homes based on internet of energy is proposed. The paper also uses energy sources to access the intelligent framework, followed by a strategy on optimization of time intervals with two different satisfaction functions. The method is based on Wi-Fi wireless technology. The Sketch up environment has been established for designing and placement of the considered home devices. The paper then used an improved version of GWO, and ABC optimization algorithms to improve the system efficiency in terms of energy consumption cost and the user’s satisfaction. The suggested platform uses Transmission Control Protocol/Internet Protocol (TCP/IP) for local microgrid data exchange and as a backup communication method among microgrid in case of a failure in the cloud level communication. MQTT subscriber /publisher is adopted for cloud-level messaging and HTTP for interactions between a cloud-server and the platform.

With implementing suggested EMS, it is notable that micro grid consumed energy cost have been minimized from 542.2977 cent to 412.9103 cent (31.335% of the operation cost) by using rainfall algorithm. The scheduling controller suggested in this paper succeeded the energy saving of 25.98% for the first home, 26.45% for the second home, 23.45% for the third home per day. Furthermore, it is notable the suggested GWO method in our work saved 19.47% for home 1, 27.14% for home 2, 23.45% for home 3, 32.5% for home 4, 24.4% for home 5 and 32.96% for home 6 per-day. In addition, it is notable the suggested ABC method in our work saved 32.75% for home 1, 41.26% for home 2, 36.22% for home 3, 52.81% for home 4, 36.11% for home 5 and 54.6% for home 6 per-day. The suggested strategy gives better efficiency to the users for participating in the demand response.

Future extension of this work may include the integration of the LoRaWAN network with the proposed IoT architecture, because the use of the LoRaWAN technology could lead to a very promising solution, due to its good coverage capabilities (both in outdoor and in hybrid environments), whereas its most critical aspect is represented by the relatively low data throughput and duty cycle limitation.

## Figures and Tables

**Table 1:** The main aspects of each control technique.

Technique	Communication Scheme	Fault Tolerance	Communication Constraints	Communication Band Width
Centralized control	One to all	Week	Robust	High
Decentralized local control	One to all	Strong	None	None
Distributed Control	All to all	Strong	Strong	Very High

**Table 2:** Contributions versus shortcomings of the most recent papers concerning energy management system.

Reference	Contributions	Shortcomings
[10]	Proposed a two-stage optimization model for optimal planning of household renewable energy resources integration with the association of customer-based EMS.	The energy management for a multi-agent system governed microgrid in Energy Internet not investigated.
[11]	Proposed a smart residential energy management system for customer of intelligent residential buildings, and its benefits are demonstrated by a case study.	The data processing and storage using IoT layer platform is not considered.
[12]	Proposed a multi-objective day-ahead system model to optimize the economy and comfort of the occupants by delivering the source load storage in a synergistic fashion.	The authors did not consider the real-time change in users’ demand where there is a chance of electricity loss whenever a user curtails his electric load demand. The multi-agent approach does not implement in this paper. The data processing and storage using IoT layer platform is not considered.
[13]	Presented an interdisciplinary approach that combines machine education, maximization and design of data structures to create a system to respond to real-life needs at home.	The multi-agent approach does not implement in this paper. The data processing and storage using IoT layer platform is not considered.
[14]	Proposed a stochastic model for the home energy management system by considering the availability uncertainties and small-scale generation of renewable energy sources.	The data processing and storage using IoT layer platform is not considered. The multi-agent approach does not implement in this paper.
[15]	Proposed chance constrained optimization to optimize the process of devices in a resident management system in an indeterminate environment.	The energy management for a multi-agent system governed microgrid in Energy Internet not investigated.
[16]	Suggested a new hierarchical energy management system based on optimization for multi-microgrid.	These studies do not consider the tradeoffs between electricity bills and user discomfort. The energy management for a multi-agent system governed microgrid in Energy Internet not investigated.
[17]	Proposed a robust optimization method for the day ahead home energy management system to reduce the energy price.	The energy management for a multi-agent system governed microgrid in Energy Internet not investigated.
[18]	Proposed more realistic and precise analytical models under four power request control scenarios for peak demand determination in a residential environment.	This study does not consider the tradeoffs between electricity bills and user discomfort. The energy management for a multi-agent system governed microgrid in Energy Internet not investigated.
[19]	Proposed a novel energy management system and control method for a smart grid system depended on green energy.	The data processing and storage using IoT layer platform is not considered. The multi-agent approach does not implement in this paper.
[20]	Presented an IoE transactive energy management systems based on fog architecture.	The multi-agent approach does not implement in this paper.
[21]	Presented an IoT based computer energy management, which generates a consumer load profile for remote access by a utility company or a consumer.	The multi-agent approach does not implement in this paper.
[22]	Proposed an IoT house energy management system for fog computing applications based on Zigbee, MQTT and Wi-Fi sensor nodes.	A cloud-based platform for multi-agents hybrid microgrid not investigated in this paper. However, the authors did not use a meta-heuristic technique (the grey wolf optimizer, artificial bee colony optimization algorithm, etc.) to minimize the cost.
[23]	Proposed a multi-objective problem, whose resolution takes place using an evolutionary algorithm and a task management methodology.	However, implementation of these systems in a real environment is costly and can in a user rang through a centralized system (i.e., cloud or fog-based system). A cloud-based platform for multi-agents hybrid microgrid not investigated in this paper.
[24]	Proposed Adaptive Energy Management System for Smart Microgrids.	The multi-agent approach does not implement in this paper. A cloud-based platform for multi-agents hybrid microgrid not investigated in this paper.
[25]	Proposed real-time optimum schedule controller for EMS using binary game theory algorithm.	The data processing and storage using IoT layer platform is not considered. The multi-agent approach does not implement in this paper.
[26]	Proposed a novel robust control method for operated parallel inverters in green applications.	The multi-agent approach does not implement in this paper. This study does not consider the tradeoffs between electricity bills and user discomfort.
[27]	Proposed a novel energy management system of on-grid/off-grid utilizing adaptive neuro-fuzzy inference framework.	The multi-agent approach does not implement in this paper. The data processing and storage using IoT layer platform is not considered.
[28]	Proposed Voltage Over-scaling-based Lightweight Authentication for IoT Security.	The multi-agent approach does not implement in this paper. This study does not consider the tradeoffs between electricity bills and user discomfort.
[29]	Proposed the first Physical Unclonable Function-based key sharing method that the same shared-key can be generated in physically for all devices so that it can be applied in the lightweight key-sharing protocol for IoT devices.	The multi-agent approach does not implement in this paper. This study does not consider the tradeoffs between electricity bills and user discomfort.
[30]	Presented a real-time demand-side management framework based on robust model predictive control (RMPC) for residential smart grids.	The energy management for a multi-agent system governed microgrid in Energy Internet not investigated.
[31]	Proposed Energy Management in Electrical Smart Grid Environment Using Robust Optimization Algorithm.	The energy management for a multi-agent system governed microgrid in Energy Internet not investigated.
[32]	Proposed a distributed demand-side management (DSM) approach for smart grids taking into account uncertainty in wind power forecasting.	The energy management for a multi-agent system governed microgrid in Energy Internet not investigated.
[33]	Proposed a robust economic model predictive control method that guarantees an optimal energy dispatch in a smart micro-grid.	The energy management for a multi-agent system governed microgrid in Energy Internet not investigated.

**Table 3:** Micro grid appliances detail description.

Type	Daily Usage	Power (kW)	Appliances
Devices of Fixed loads	24	0.25	Refregrator
	5	3	HVAC
	8	0.2	TV
	9	0.25	Lights
	18	0.3	PC
	24	0.1	Cameras
	3	1	Oven
Devices of Non shiftable loads	3	1.5	Washing machine
	4	1	Clothes Dryer
	2	1	Dish washer
	2	1.2	Electric frying pot
Devices of Shiftable loads	6	1.5	Water heater
	3	1.7	Vacuum cleaner
	4	1	Water pump
	2	3.5	Gayser

**Table 4:** Difference between with and without corrective method.

Type	Home 1	Home 2	Home 3	Home 4	Home 5	Home 6
Cost without corrective method (cent)	182.07	183.76	177.4667	184.0955	190.8146	182.13
Cost with GWO corrective method (cent)	152.386	144.5312	143.7466	138.9	153.3869	136.98
Percentage improvement using GWO corrective method	19.47%	27.14%	23.45%	32.5%	24.4%	32.96%
Cost with ABC corrective method (cent)	137.1482	130.078	130.27	120.4749	136.19	117.8039
Percentage improvement using ABC corrective method	32.75%	41.26%	36.22%	52.81%	36.11%	54.6%

## Data Availability

Data sharing not applicable.
